# Safety and efficacy of oral DMSA therapy for children with autism spectrum disorders: Part A - Medical results

**DOI:** 10.1186/1472-6904-9-16

**Published:** 2009-10-23

**Authors:** James B Adams, Matthew Baral, Elizabeth Geis, Jessica Mitchell, Julie Ingram, Andrea Hensley, Irene Zappia, Sanford Newmark, Eva Gehn, Robert A Rubin, Ken Mitchell, Jeff Bradstreet, Jane El-Dahr

**Affiliations:** 1 Division of Basic Medical Sciences, Southwest College of Naturopathic Medicine, Tempe, AZ, USA; 2 Department of Pediatric Medicine, Southwest College of Naturopathic Medicine, Tempe, AZ, USA; 3 Autism Research Institute, San Diego, CA, USA; 4 Center for Integrative Pediatric Medicine, Tucson, AZ, USA; 5 Department of Mathematics, Whittier College, Whittier, CA, USA; 6 International Child Development Resource Center, Phoenix, AZ, USA; 7 Department of Pediatrics, Tulane University School of Medicine, New Orleans, LA, USA

## Abstract

**Background:**

This study investigated the effect of oral dimercapto succinic acid (DMSA) therapy for children with autism spectrum disorders ages 3-8 years.

**Methods:**

Phase 1 involved 65 children who received one round of DMSA (3 days). Participants who had high urinary excretion of toxic metals were selected to continue on to phase 2. In phase 2, 49 participants were randomly assigned in a double-blind design to receive an additional 6 rounds of either DMSA or placebo.

**Results:**

DMSA greatly increased the excretion of lead, substantially increased excretion of tin and bismuth, and somewhat increased the excretion of thallium, mercury, antimony, and tungsten. There was some increase in urinary excretion of essential minerals, especially potassium and chromium. The Phase 1 single round of DMSA led to a dramatic normalization of RBC glutathione in almost all cases, and greatly improved abnormal platelet counts, suggesting a significant decrease in inflammation.

**Conclusion:**

Overall, DMSA therapy seems to be reasonably safe, effective in removing several toxic metals (especially lead), dramatically effective in normalizing RBC glutathione, and effective in normalizing platelet counts. Only 1 round (3 days) was sufficient to improve glutathione and platelets. Additional rounds increased excretion of toxic metals.

## Background

Autism is a severe developmental disorder which involves social withdrawal, communication deficits, and stereotypic/repetitive behaviors. The cause(s) of autism are unknown, but both genetic and environmental factors have been implicated. One environmental factor that has received significant attention is the body burden of mercury and other toxic metals.

Heavy metal toxicity can occur from either a high exposure or a decreased ability to excrete heavy metals, with the latter case seeming to be the primary issue in autism. The primary mechanism for excreting mercury and some other toxic metals from the body involves binding to glutathione and then being excreted in the bile [1]. Infants are especially vulnerable to metal poisoning because they are poor excretors due to low production of glutathione [2], and because they are usually on all-milk diets, which decreased mercury excretion by a factor of three in a study of rats [3].

Infants with autism are even more vulnerable to mercury and other heavy metals, because their glutathione levels are much lower than those of typical children [4-6] and a higher fraction of their glutathione is oxidized [4,5]. Also, four studies have found that children with autism had much higher usage of oral antibiotics than typical children [7-10]. High oral antibiotic usage is concerning because previous rat studies found that oral antibiotics resulted in a near-total loss of the ability to excrete mercury [3,11]. The reason appears to be that normal gut anaerobes are able to convert methylmercury (which is rapidly absorbed) into inorganic mercury (which is poorly absorbed and hence mostly excreted). In contrast, most strains of yeast and E. coli carry out the reverse reaction, namely the methylation of inorganic mercury to methylmercury [12]. So, high oral antibiotic use leads to a loss of normal gut flora and an increase in yeast and E. coli, resulting in a loss of ability to demethylate methylmercury and an increased methylation of inorganic mercury, ultimately resulting in decreased excretion and increased uptake of mercury.

A decreased ability to excrete mercury is consistent with a study [13] which found that children with autism had only 1/8 the normal amount of mercury in their baby hair compared to typical infants, even though both groups had a similar exposure to the major sources of mercury (maternal seafood, mercury dental amalgams, and thimerosal in vaccines). There was also a significant inverse correlation in the severity of autism and the level of mercury in the hair, suggesting that the children with the weakest ability to excrete mercury developed the most severe symptoms. These results together suggest a decreased ability to excrete mercury, resulting in a higher body burden. A replication study [10] also found that children with autism tended to have lower levels of mercury in their baby hair than did typical infants. A recent study [14] of children ages 1-5 years found lower levels of lead, cadmium, and arsenic in the hair of children with autism, suggesting a decreased ability to excrete those metals; there was a similar trend for mercury, but it was not statistically significant in those older children.

There have also been studies of the level of mercury in the hair and blood of older children with autism. These studies are of limited relevance because mercury has a half-life of only several weeks in the blood, so they do not reflect levels in early infancy when autism developed. One study in the US found elevated levels of mercury and other toxic metals in the blood [6]. A study in Hong Kong found a possible difference in blood levels (p = 0.056) but not in hair [15]. However, a re-analysis of their data found an abnormal ratio of mercury in blood to mercury in hair, suggesting an excretion problem [16]. One study in the US did not find any difference in the level of mercury in the hair of older children (ages 3-15 years) with autism vs. controls [17].

A decreased ability to excrete mercury should result in a higher body burden, and that was demonstrated in a study which investigated the effect of giving DMSA (dimercaptosuccinic acid) to 221 children with autism compared to 18 controls [18]. They found that the children with autism excreted 3.1 times as much mercury into their urine (which is where DMSA is excreted), p < 0.0002, but lead and cadmium levels were not significantly different. Similarly, a small study [9] found that children with autism had a 2× higher level of mercury in their baby teeth compared to typical children. DMSA provocation testing is probably a measure of both recent and older exposures, whereas baby teeth are a measure of pre-natal and infantile exposure.

A previous study [8] investigated possible factors affecting heavy metal exposure in the medical history of children with autism vs. controls. Four exposures were found to be statistically different in children with autism spectrum disorders (ASD) (n = 53) vs. controls (n = 48), including higher maternal seafood consumption (RI = 2.7, p = 0.02); higher otitis media-related antibiotic use (RI = 2.5, p = 0.0001); more eating/licking paint (p = 0.04), and more pica (30% vs 2%, p = 0.0002). The ASD children also had more immediate adverse reactions to vaccinations (RI = 2.5, p = 0.002). The dental histories did not reveal major differences, although there was a trend that placement or removal of mercury fillings during pregnancy was more common in the mothers of children with ASD than the controls (R.I. = 7.0, p = 0.08).

There have been many epidemiological studies of the possible link between thimerosal-containing vaccines and autism since it was discovered in 1999 that the amount of thimerosal in US childhood vaccines was far in excess of FDA and EPA guidelines. Many studies by one group reported a significant link [19-29], while 5 studies by other groups failed to establish a link, [30-34], and one study was inconclusive [35]. So, the link of autism to thimerosal remains controversial.

A review article by Bernard et al. [36] reported that all of the major symptoms of infantile autism are also commonly observed in infantile mercury poisoning, including mental symptoms (language deficits, stereotypic behaviors, social withdrawal) and physical symptoms (gastrointestinal problems, sleep problems, low muscle tone, and excessive salivation). A review by Nelson and Bauman [37] disputes the Bernard et al. [36] paper, and argues that the symptoms and neuropathology of autism are distinct from mercury poisoning. However, the Toxicological Profile of Mercury published by the US Agency for Toxic Substances and Disease Registry [38] states that infantile exposure to mercury can result in neurological damage, mental retardation, incoordination, muscle weakness, seizures, and inability to speak, which are similar to the symptoms of autism.

One study [39] found elevated urinary precoproporphyrins in children with autism (n = 106) vs. controls (n = 107), which is indicative of the effect of mercury on heme synthesis. They found that those levels decreased significantly after several months of DMSA therapy. Two smaller studies [40,41] also found that children with autism had higher levels of coproporphyrins than did controls, and the children with autism had higher levels than the children with PDD/NOS or Asperger's. One of the studies [41] also found elevations in precoproporphyrins. Both studies found that children with autism who had undergone chelation therapy had less elevation in their coproporphyrin levels. Another study [42] found that the level of mercury-related porphyrins (coproporphyrin and precoporphyrin) had a significant correlation with their severity of autism as rated by the Childhood Autism Rating Scale (CARS). A recent report [43] found that the use of DMSA could improve the learning, attention, and arousal regulation of lead-exposed animals. However, that same paper also found that high-dose administration of DMSA could cause lasting cognitive damage in rats that were not lead exposed, so DMSA should only be used when lead or similar toxic metals are present.

Overall, there is a substantial body of research that suggests mercury and other toxic metals could be involved in the etiology of some cases of autism, although the autism-thimerosal link remains controversial. There is also more general evidence to suggest that lead, mercury, and other toxins can impair child development at levels commonly encountered by most of the US population [44]. There is some evidence to suggest that the use of DMSA therapy to remove toxic metals may be helpful in normalizing the porphyrin-heme pathway, and possibly helpful in improving some of the symptoms of autism.

The purpose of this paper is to evaluate the safety and efficacy of DMSA therapy on children with autism spectrum disorders (ASD), focusing on medical effects. The effects of DMSA on behavior are discussed in the following paper [45]. Also, this study found a high correlation of the severity of autism with the urinary excretion of toxic metals, and the results are reported in another paper [46]. DMSA is FDA-approved for the treatment of lead poisoning in children as young as 2 years, and this study investigated its use for an off-label application, namely treating children with ASD who have evidence of significant heavy metal exposure (based on urinary excretion after DMSA challenge). DMSA preferentially binds to lead, but can also increase the excretion of several other toxic metals (including mercury) to a lesser extent. Other agents, such as DMPS, have a stronger affinity for mercury, but they are not FDA-approved for human use, so it was decided to begin an initial detoxification study with DMSA.

The specific objectives of this study include:

1) Evaluating the effect of DMSA therapy on children with ASD, including effect on excretion of toxic metals, excretion of essential minerals, level of glutathione, blood chemistry, complete blood count (CBC), and symptoms of autism.

2) Investigate possible correlations of the severity of autism with urinary excretion of toxic metals and initial level of glutathione.

## Methods

**Figure 1:**
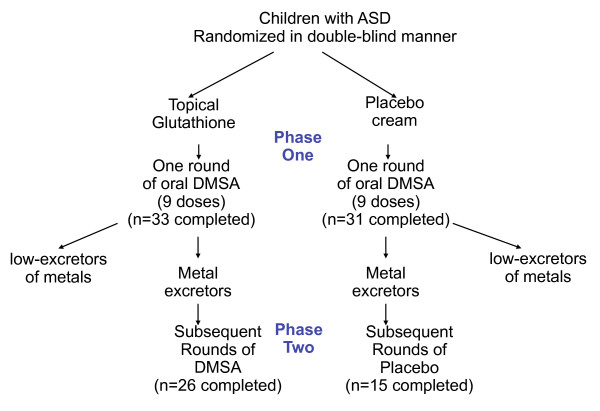
**Study design**. Both groups of participants received a single challenge round of oral DMSA; those excreting significant heavy metals continued on to Phase 2, and received additional 6 additional rounds of DMSA or placebo.

The study was designed with one screening round (9 doses over 3 days) of DMSA in Phase 1, followed by a randomized, double-blind, placebo-controlled study of 6 additional rounds (see Figure 1). The purpose of the screening round was to only allow participants with high excretion of toxic metals into phase 2. However, as discussed below, the single screening round of DMSA had an unexpectedly dramatic effect on improving abnormal glutathione and platelet levels, and that effect lasted until the end of phase 2, so that phase 2 was not a placebo-controlled investigation. Instead, it appears best to interpret this study as a comparison of the effect of 1 round of DMSA (and 6 rounds of placebo) vs. 7 rounds of DMSA. In discussing these two groups below, we will refer to them as the "1 round" group and the "7 round" group.

### Participant Selection

This study was conducted with the approval of the Human Subjects Institutional Review Board of Southwest College of Naturopathic Medicine. All parents and (where possible) children signed informed consent/assent forms.

The criteria for **Phase One **were:

1) Children with autism spectrum disorder, diagnosed by a psychiatrist, psychologist, or developmental pediatrician

2) Age 3-8 years

3) No mercury amalgam dental fillings

4) No previous use of DMSA or other prescription chelators

5) No anemia or currently being treated for anemia due to low iron.

6) No known allergies to DMSA

7) No liver or kidney disease

8) Children are well-hydrated (receiving adequate daily intake of water)

The criteria for **Phase Two **were:

1) Excretion of high amounts of toxic metals in phase one (defined below)

2) Normal liver function (serum transaminases ALT and AST), normal renal function (although low creatinine was allowed), and complete blood cell count was not below the normal reference range.

3) No changes in medication, supplements, diet, or behavioral interventions during the study.

4) At least a two-month history of taking a multi-vitamin/mineral supplement with at least the RDA of zinc, and continuing to take that during Phase Two.

5) Continue to stay well-hydrated (receive adequate daily intake of water).

The characteristics of the participants are given in Table 1. All participants were Arizona residents.

**Table 1:** Characteristics of Participants

	**Total**	**Phase 2 - treatment**	**Phase 2 - placebo**
**Total Participants**	77	26	15
**Male**	69	24	14
**Female**	8	2	1
**Age (years)**	6.3	6.7	6.5
**Diagnosis**	95% autism, 3% PDD/NOS, 3% Asperger's	96% Autism, 4% Aspergers	100% Autism

### Methodology

#### Phase One

1) Each child first received a physical examination to determine that he/she was in sufficiently good health to participate in the study.

2) A blood draw was conducted to test for blood chemistry (including kidney and liver function) and Complete Blood Count (CBC), and Red Blood Cell (RBC) glutathione. If liver and renal function were normal and CBCs were not below the normal range, then the child was eligible to participate in Phase 1.

3) Each parent filled out an initial Autism Treatment Evaluation Checklist (ATEC) questionnaire and Heavy Metal Exposure Questionnaire.

4) Parents collected a baseline first-morning urine sample.

5) Parents began daily administration of a lotion that contained glutathione or placebo, administered in a randomized, double-blind fashion. It was administered 1×/day, after bath on clean skin, with the child rubbing it on. Glutathione was administered to half the children to see if it affected their excretion of toxic metals and their levels of toxic metals.

6) After 7 days of "glutathione" lotion, all children received oral DMSA. The dosage was 10 mg/kg-dose, 3×/day, for 3 days. The DMSA was compounded individually for each child from pharmaceutical grade DMSA (over 99% pure) supplied by Spectrum Chemical. Parents collected all urine for approximately 8 hours after the first dose and for approximately 8 hours after the 9th dose. Children were encouraged to urinate prior to taking the 1^st ^and 9^th ^dose, but compliance was not required.

7) Urine samples were frozen and shipped to Doctor's Data Laboratories (St. Charles, IL) for measuring excretion of toxic and essential minerals. Children with high excretion of toxic metals (arbitrarily defined as above Doctor's Data reference range, defined as the top 95% for typical children who are not undergoing chelation therapy) were allowed to continue on to Phase 2. Note that this level was somewhat arbitrary, and was chosen so that only children with significant toxic metal excretion would enter phase two.

#### Phase Two

1) The blood chemistry, CBC, and RBC glutathione were re-measured at the beginning of phase 2. If liver and renal function were normal, if CBC's were not low, and if there was significant excretion of metals in Phase I, then the children were selected to continue into phase two

2) Autism Diagnostic Observation Schedule (ADOS) examinations were administered (approximately 1 hour assessment) by an ADOS-certified evaluator to further characterize the participants. Each child was always examined by the same evaluator at the beginning and end. Of the 41 participants that were evaluated and finished the study, 81% met the criteria for Autism, 12% more met the criteria for ASD, and 7% did not meet the criteria for ASD, according to the sum of the Communication + Social Scores. All children continued on in the study, since they all had a previous clinical diagnosis of ASD.

3) Parents filled out two questionnaires on severity of autism, the Pervasive Developmental Disorders - Behavior Inventory (PDD-BI) and Severity of Autism Scale (SAS).

4) Children received the DMSA or placebo, and took them for up to 3 rounds. Each round consisted of 3 days of DMSA/placebo (as in Phase 1), followed by 11 days of no DMSA/placebo. The children who had originally received glutathione lotion were given DMSA, and those who had received the placebo "glutathione" lotion received the placebo "DMSA". The families continued to be blinded in the second phase of the study. This design resulted in one group of children who had topical glutathione and oral DMSA challenge followed by continued administration of DMSA and a second group of participants who underwent a single round of DMSA challenge without topical glutathione and who subsequently did not receive any further chelation.

5) Urine was collected after the 9^th ^dose of DMSA (or placebo) at the end of the 2^nd ^round in Phase 2. This was shipped to Doctor's Data for testing.

6) After the 3^rd ^round of DMSA (or placebo) in Phase 2, blood chemistry and CBC were measured again. If children had normal kidney/liver function and normal CBC, and if they continued to have elevated excretion of toxic metals in their urine based on their 2^nd ^round of testing, they were given another 3 rounds of DMSA/placebo.

7) After the 6^th ^round of DMSA in Phase 2, a final blood chemistry and CBC were measured.

8) When the participants finished Phase 2 (after either 3 or 6 rounds), an ADOS evaluation was conducted by the same evaluator who conducted the first evaluation. The parents again filled out the ATEC, SAS, and PDD-BI, as well as the Parental Global Impressions questionnaire. The professional working with the child was asked again to fill out the ATEC form for the child.

### Dosage of DMSA

The dosage of oral DMSA was 10 mg/kg per dose, in 3 doses per day. This is the dosage recommended in the Physician's Desk Reference (PDR), and the dosage we used in our previous 9-dose study [18].

### Placebo

The placebo was methyl cellulose, an inert material commonly used as a filler. It was packed in identical pills and bottles as the DMSA. Since DMSA has a strong smell, each bottle included a small slotted container that contained DMSA, so that the medication smell was present in the container.

#### Dosage of Glutathione

The daily topical dosage of glutathione was approximately 180 mg of reduced l-glutathione in a lotion of isopropyl myristate, mineral oil, caprylic/capric trigliceride, and vitamin E acetate. The placebo was identical in packaging and formulation except it did not contain glutathione.

#### Chemistry Panel and Complete Blood Count (CBC)

Measured by Quest Laboratories.

#### Urine Testing

Toxic and essential minerals were measured by Doctors' Data, using Inductively-Coupled Plasma -Mass Spectrometry, and creatinine was measured using the Jaffe method. The laboratory was blinded to treatment and placebo groups.

### Red Blood Cell (RBC) Glutathione

Most glutathione in whole blood is found in the erythrocyte. RBC glutathione was measured by Immunosciences Lab using a kit provided by Oxis Research (Foster City, CA). The method is based on the formation of a chromophoric thione. The absorbance measured at 405 nm is directly proportional to the GSH concentration.

### Statistical Analysis

Several types of statistical analyses were used, depending on the research question being addressed. Pearson Correlation coefficients were obtained to determine the strengths of linear relationships among the variables involved in the analyses. In comparing pre/post levels within a group (such as change in urinary metal excretion or changes in a participant's autism severity), 2-sided paired t-tests were used, and for each individual hypothesis a p value of 0.05 or lower was assumed significant for individual analyses. However, when multiple comparisons were considered, then a Bonferroni correction was used.

Since this study is largely exploratory, we examined the relationships among several of the measured quantities, for example the relation between changes in the various autism severity measurement and changes in excretion levels of toxic metals after the first dose of DMSA in phase 1, or the relationship between initial glutathione levels and initial toxic metals excretion levels in subjects prior to the administration of the first dose of DMSA in Phase 1. For the selected dependent and independent variables, step-wise linear regression analyses were conducted; at each step, the variable with the highest p-value was eliminated, and this process was continued until the adjusted R^2 ^value began declining. Thus, the goal was to determine the best fit to the database for the selected model, taking into account the correlation among the independent variables. Since the database had a few missing values (due to missing lab or behavioral data), the regression analyses were conducted in two slightly different ways which generally yielded very similar results: 1) eliminate all participants with missing data for any of the variables in the model at the beginning of the analysis, and 2) eliminate participants on an as-needed basis (i.e., only where there is missing data for any variable in the current step in the analysis). Since these two methods generally yielded very similar results, we usually only report the results for method 1). The only exception was for the change in the ADOS, in which case we report the results of both methods.

### Participation

#### Phase 1 Participation

We began with 82 participants, enrolled on a rolling basis. 77 completed the initial blood draw, and 65 completed Phase 1. Of the initial 82:

One did not qualify for the study due to elevated liver enzymes. Four stopped after the physical examination. Eleven stopped after the initial blood draw (in two cases this was due to difficulty in collecting urine samples). One participant took DMSA and collected a urine sample, but did not send it to the lab due to lack of interest/time.

#### Participant Transition to Phase 2 and Adverse Events

Of the 65 children who completed Phase 1, 49 continued to phase 2. Nine children were not allowed to continue to Phase 2; in 8 cases this was due to low urinary excretion of toxic metals, and in 1 case it was due to extremely high lead excretion (both at baseline and after DMSA). (This patient was referred for follow up to their primary care physician for evaluation of acute lead exposure). Another 7 families did not continue for personal reasons unrelated to the study.

Finally, there was one case of a mild adverse reaction (lethargy, decreased appetite) during Phase I, and that family decided not to continue into Phase 2.

#### Phase 2 Participation

49 children began Phase 2, and 41 finished (including 5 in treatment group who finished early due to low excretion of urinary toxic metals after 2^nd ^round of DMSA in Phase 2.

• 1 was discontinued due to elevated liver enzymes due to their psychiatric medication.

• 1 dropped due to a death in the family

• 2 dropped due to perceived lack of benefit - 1 was on placebo, and 1 was on DMSA.

• 4 dropped due to adverse effects:

sleep problems (on DMSA)

behavior and some skills worsened (on DMSA)

behavior worsened, more self-stimulatory behavior - (on placebo)

behavior worsened, some regression (on placebo)

In most cases adverse symptoms were temporary and resolved within a week after stopping treatment. In one case of a child on DMSA, the child had a history of gaining and losing skills, and the parents thought that the child did permanently lose some skills during the study. However, the ADOS scores on that child were either unchanged (Communication, Sociability) or improved (Play: 2 to 1, Stimulatory Behavior/Restricted Interests: 4 to 2) from the beginning to the end of the study.

5 participants completed Phase 2, Round 2, but their urinary excretion of toxic metals had decreased below our cut-off and did not qualify them to continue into the rest of Phase 2; so, they completed the study after receiving 1 round of DMSA in Phase 1 and 2-3 rounds in Phase 2.

## Results

### Urinary Excretion - Toxic Metals

One participant's results were excluded from the general analyses in this section and elsewhere because she had extremely high levels of many toxic metals both at baseline and after taking DMSA, including extremely high baseline aluminum (63× average of other participants), antimony (45× average), bismuth (40× average), cadmium (7× average), lead (12× average), tin (12× average) and uranium (65× average). After DMSA, her excretion of antimony increased to 24× her baseline (1000× the average baseline), lead increased to 2.4× her baseline, and excretion of other metals was less affected. This participant did not continue into phase 2 so that she could pursue private treatment.

**Figure 2:**
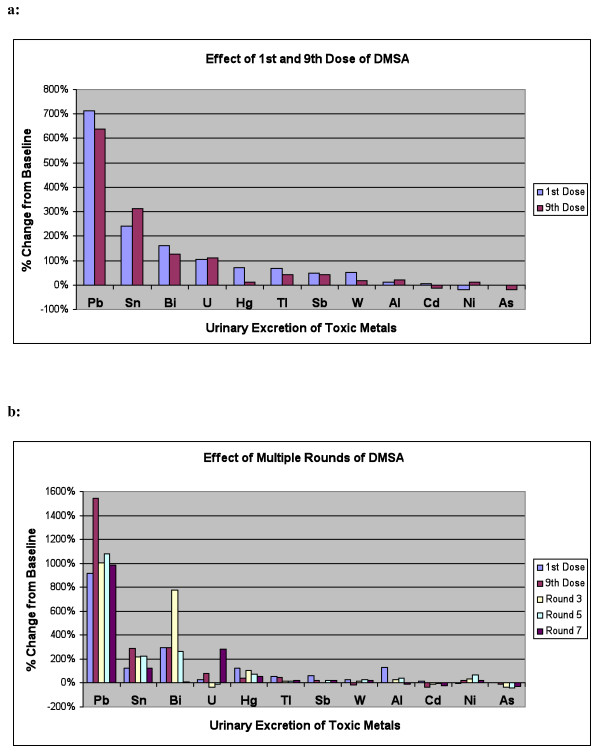
**Percentage change in urinary excretion of toxic metals**. a: Percentage Change in Urinary Excretion of Toxic Metals after 1^st ^and 9^th ^Dose of DMSA in Phase 1. (n = 63). b: Percentage Change in Urinary Excretion of Toxic Metals after 1^st ^and 9^th ^dose of Round 1, and then 9^th ^dose of Rounds 3, 5, and 7, for the group who received 7 rounds of DMSA. (n = 20).

For all other participants, Table 2 and Figure 2a shows the level of urinary excretion of toxic metals (normalized per gram creatinine) at baseline (beginning of Phase 1, before taking any DMSA), and after the first and 9^th ^doses of DMSA in the Phase 1 challenge round. The metals are listed in order of effect of DMSA on excretion. There was a very large and extremely statistically significant increase in excretion of lead. There were also large and significant increases in excretion of tin and bismuth. There was a large increase in excretion of uranium, but it was not statistically significant (only 14-15 participants had detectable excretion of uranium before or after taking DMSA). There was a large and highly significant initial increase in excretion of mercury, but at the 9^th ^dose the excretion was only slightly higher than baseline and not statistically significant. There were significant increases in excretion of thallium, antimony, and tungsten. There was little change in levels of aluminum or cadmium. There was a small initial decrease in excretion of nickel, and a small decrease in excretion of arsenic at the 9^th ^dose only. Due to multiple comparisons (12 metals), only p-values below 0.004 should be considered significant, and p values between 0.05 and 0.004 should be considered marginally significant.

**Table 2:** Urinary excretion of toxic metals in Phase 1, at baseline, after 1^st ^dose, and after 9^th ^dose, in mcg/g creatinine.

**Element**	**Baseline**	**1**^**st **^**Dose**	**9**^**th **^**Dose**	**1**^**st **^**dose vs. baseline**	**9**^**th **^**dose vs. baseline**
**Pb**	1.3 +/- 2.3	10. +/- 19.	9.2 +/- 7.8	713%***	638%***
**Sn**	2.3 +/- 3.4	8.0 +/- 13	9.7 +/- 24	241%***	314%*
**Bi**	0.18 +/- 0.45	0.47 +/- 1.4	0.41 +/- 1.0	**161%+**	128%*
**U**	0.015 +/- .04	0.036 +/- .09	0.031 +/- .1	104%	111%
**Hg**	0.86 +/- .92	1.5 +/- 1.4	0.97 +/- 0.88	70%**	**13%**
**Tl**	0.15 +/- 0.12	0.25 +/- 0.18	0.21 +/- 0.19	67%***	42%**
**Sb**	0.10 +/- 0.10	0.15 +/- 0.19	0.14 +/- 0.20	49%*	**42%+**
**W**	0.39 +/- 0.29	0.59 +/- 0.63	0.46 +/- 0.50	51%**	18%*
**Al**	16 +/- 21	18 +/- 23	19 +/- 33	11%	21%
**Ni**	6.7 +/- 5.1	5.5 +/- 3.6	7.6 +/- 4.3	-18%*	12%
**Cd**	0.38 +/- 0.24	0.40 +/- 0.24	0.33 +/- 0.23	5%	-14%
**As**	32 +/- 20	32 +/- 18	25 +/- 18	0%	-19%*
**creatinine**	94 +/- 52	62 +/- 39	80 +/- 43	-34%***	-15%*

+ P < 0.1

*P < 0.05

** P < 0.01

*** P < 0.001

Creatinine values have units of mg/dl. N = 63. The metals are listed in approximate order of effect of DMSA on excretion. Significant results are highlighted in bold font.

The effect of the glutathione lotion on excretion of toxic metals was analyzed by comparing the groups receiving the DMSA + glutathione lotion vs. DMSA + placebo lotion. In general there were no statistically significant differences in the two groups when comparing the average excretion after dose 1 plus after dose 9 vs. baseline. However, the excretion of mercury was possibly higher (p = 0.06) for the group receiving the DMSA + glutathione (average excretion of 1.38 vs 0.91, vs. baseline of 0.73 and 0.80, units of mcg/g creatinine, respectively).

Table 3 and Figure 2b shows the data for urinary excretion of toxic metals for the participants who were in the treatment group in Phase 2 (n = 20). The table lists both their Phase 1 results (1^st ^and 9^th ^dose) and their Phase 2 results (after round 2, 4, and 6 of DMSA in Phase 2). Table 3 lists the metals in the same order as in Table 2 for convenience. Note that the number of participants in this group is smaller than in Table 2 (n = 20 vs. n = 63, respectively), which affects which results are statistically significant. In general, the results for phase 2 were similar to those for phase 1. Overall, there was a very large increase in excretion of lead, some increases in excretion of tin, bismuth, mercury, and thallium, possible ncreases in excretion of uranium, antimony, tungsten, nickel, and decreases in excretion ofi cadmium and arsenic. The increased excretion of lead persisted throughout the entire study, and mercury excretion also generally continued with some fluctuations.

**Table 3:** Comparison of change in average urine excretion compared to baseline for participants who completed six rounds of phase 2 (n = 20).

**Element**	**Baseline (mcg/g- creatinine)**	**Change after 1**^**st **^**dose**	**Change after 9**^**th **^**dose**	**Change after round 2**	**Change after round 4**	**Change after round 6**
**Pb**	0.64	935%***	1562%***	1001%***	1063%***	1005%***
**Sn**	3.5	118%*	269%	205%	213%	115%
**Bi**	0.06	312%	296%	787%	261%	11%
**U**	0.014	29%	79%	-32%	-11%	**282%+**
**Hg**	0.85	120%*	37%	98%***	70%	**59%+**
**Tl**	0.16	54%**	44%*	13%	18%	28%
**Sb**	0.10	**67%+**	26%	9%	23%	27%
**W**	0.41	**36%+**	-11%	14%	52%	25%
**Al**	8.3	125%	1%	26%	38%	-12%
**Ni**	5.8	-2%	25%	**36%+**	68%*	25%
**Cd**	0.35	14%	-32%*	-7%	0%	-17%
**As**	31	4%	-13%	-39%***	-42%***	**-31%+**

+ P < 0.1

*P < 0.05

** P < 0.01

*** P < 0.001

Significant results are highlighted in bold font.

Table 4 lists the correlations of urinary metal excretion with one another at baseline, Table 5 lists the correlations at 9^th ^dose of Phase 1, and Table 6 lists the correlations at 9^th ^dose vs. Baseline. Most of the metals have one or more significant correlations with other metals, so that regression analysis had some difficulty in distinguishing the effect of one metal from another. Since multiple comparisons were made, only p values less than 0.01 are listed.

**Table 4:** Significant Correlations of Baseline Urinary Metal Excretion with Baseline Urinary Metal Excretion (only correlations >0.31 in magnitude are listed, corresponding to p = 0.01 on an individual comparison basis)

**Metal**	**Correlations**
**Pb**	Tl (0.37), As (0.44), Al (0.41)
**Sn**	Tl (0.36), Sb (0.34)
**Hg**	Sb(0.42)
**Tl**	Sn (0.36)
**Sb**	Sn (0.34), Hg (0.42)
**W**	none
**Al**	Pb(0.41)
**Cd**	none
**As**	Pb(0.44)

**Table 5:** Significant Correlations of Phase 1, 9^th ^dose Urinary Metal Excretion with Phase 1, 9^th ^dose Urinary Metal Excretion (only correlations >0.31 in magnitude are listed, corresponding to p = 0.01 on an individual comparison basis)

**Metal**	**Correlations**
**Pb**	Tl(0.47), Sb(0.35), Al (0.56)
**Sn**	none
**Hg**	none
**Tl**	Pb(0.47)
**Sb**	Pb(0.35), Cd(0.67), Al (0.63)
**W**	None
**Al**	Pb (0.56), Sb (0.63), Cd (0.67)
**Cd**	Sb (0.67), Al (0.67)
**As**	Pb (0.56), Sb (0.63), Cd (0.67)

**Table 6:** Significant Correlations of Phase 1, 9^th ^dose Urinary Metal Excretion with Baseline Urinary Metal Excretion (only correlations >0.31 in magnitude are listed, corresponding to p = 0.01 on an individual comparison basis)

**Metal at 9**^**th **^**dose**	**Correlations with baseline excretion**
**Pb**	Pb(0.40), Tl(0.51),
**Sn**	W(0.38)
**Hg**	none
**Tl**	Tl(0.54), W(0.31)
**Sb**	Pb(0.81), Tl(0.34), As(0.40), Al(0.38)
**W**	None
**Al**	Pb(0.59), As(0.33), Al(0.35)
**Cd**	Pb(0.62), As(0.42), Cd(0.38), Al (0.40)
**As**	Pb(0.59), As(0.33), Al(0.35)

### Urinary Excretion of Essential and Other Minerals

**Figure 3:**
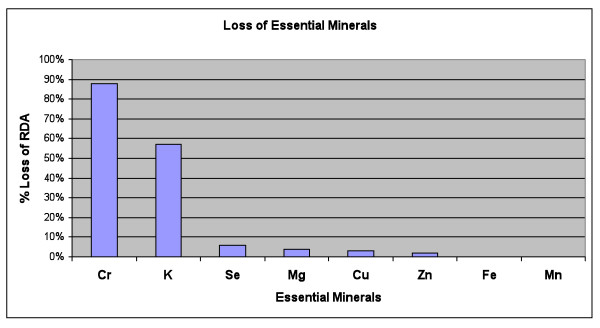
**Loss of Essential Minerals, averaged over the 3 days of DMSA therapy in Phase 1, in units of the % RDA**. The only major losses are chromium and potassium.

Table 7 and Figure 3 show the average urinary excretion of essential and other metals for Phase 1, including the values at baseline, and the percentage change after the 1^st ^dose of DMSA and after the 9^th ^dose of DMSA in Phase 1. There was a large and statistically significant excretion of copper, potassium, and manganese. Excretion of iron increased and was marginally statistically significant. There was a moderate initial increase of excretion of vanadium, chromium, sodium, and boron, but then they were similar to baseline by the 9^th ^dose. The excretions of zinc and magnesium were initially similar to baseline and then had a moderate increase by the 9^th ^dose. There were large average increases in zirconium and lithium (due to a few outliers), but the increases were not statistically significant. The excretion of barium, sulfur, selenium, cobalt, calcium, strontium, and phosphorus were not significantly affected. There was a moderate decrease in the excretion of molybdenum at the 9^th ^dose which was marginally significant. Since multiple comparisons were made, only p values below 0.003 should be considered significant.

**Table 7:** Comparison of average urine excretion of essential and other minerals compared to baseline.

	**Baseline**	**st dev**	**Change after 1**^**st **^**dose**	**Change after 9**^**th **^**dose**	**RDA for 4-8 yr (mg)**	**% of RDA lost #**
**Cu**	0.022	0.008	74%***	129%***	0.44	3%
**K**	2187	1491	110%***	48%**	1500	**57%**
**Fe**	0.016	0.055	93%+	64%	10	0.1%
**Mn**	0.0023	0.0016	77%***	37%*	1.5	<0.1%
**Zi**	0.00034	0.00069	90%	13%	n/a	
**Li**	0.10	0.10	51%	50%	n/a	
**V**	0.025	0.013	49%***	10%		
**Cr**	0.091	0.044	47%***	11%	0.015	**88%**
**Zn**	0.81	0.49	8%	30%***	5	2%
**Na**	3901	2018	35%***	3%	n/a	
**B**	2.5	2.0	25%*	11%	n/a	
**Ba**	0.0050	0.0052	12%	22%	n/a	
**S**	1140	694	3%	9%	n/a	
**Mg**	168	64	-5%	17%*	130	4%
**Se**	0.20	0.16	3%	0%	30	6%
**Co**	0.0014	0.0013	-4%	4%	n/a	
**Ca**	137	115	-6%	3%	800	no extra loss
**St**	0.25	0.15	-5%	0%	n/a	
**P**	1160	627	-5%	-4%	500	No extraloss
**Mo**	0.13	0.13	-5%	-33%*	0.022	No extra loss

+ P < 0.1

*P < 0.05

** P < 0.01

*** P < 0.001

# Averaged over 3 days, assuming production of creatinine is 500 mcg/day, which is typical for a 60-pound child

N = 63. Units are mg/g-creatinine (note difference from table 2 and 3, which are mcg/g-creatinine). Arranged in approximate order of increase in urinary excretion. The extra loss of minerals due to increased urinary excretion is also listed in terms of the RDA. Results for potassium (K) and chromium (Cr) are highlighted due to the large % of RDA.

Table 7 also lists the extra loss of essential minerals, in terms of the fraction of the Recommended Daily Allowance (RDA) lost each day, based on two reasonable assumptions: 1) averaging the loss between the 1^st ^and 9^th ^dose, and 2) assuming a daily urinary excretion of 500 mcg of creatinine, which is typical of a 60-pound child. On that basis, there was a large daily loss of chromium (88% of the RDA) and potassium (57% of the RDA). The amount of loss of other essential minerals was negligible (less than 6% of the RDA).

It should be noted that all the participants were taking a vitamin/mineral supplement for at least two months prior to and throughout the study, to minimize effects due to possible loss of essential minerals.

### RBC Glutathione

**Figure 4:**
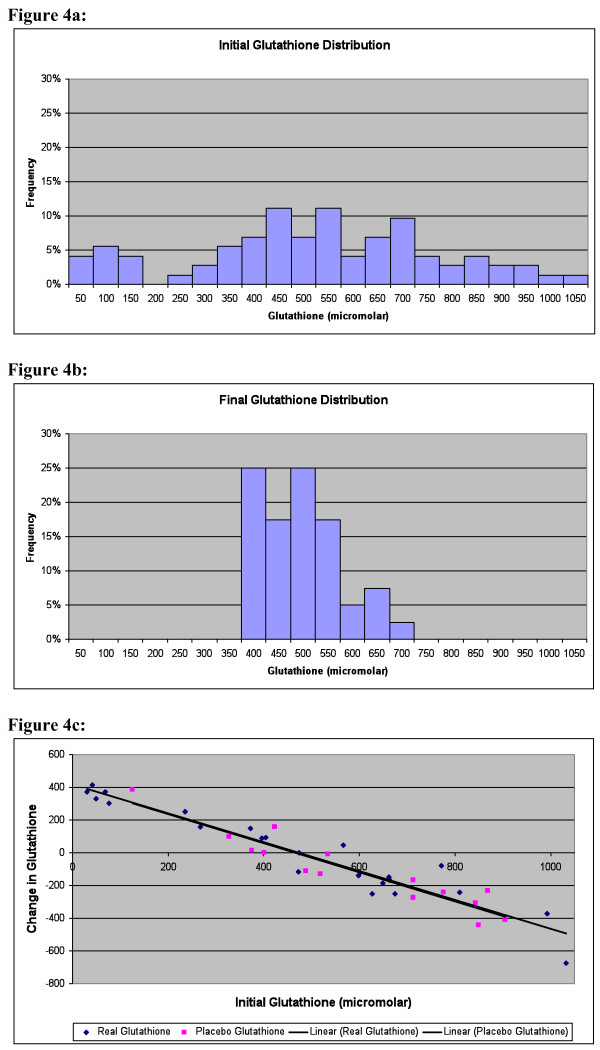
**RBC glucathione distributions**. **a: Initial RBC Glutathione Distribution (pre-treatment)**. Many autistic children have levels below and above the laboratory's reference range of 427-714 micromolar. Each histogram corresponds to the frequency between that value and 50 below it; ie, the histogram labelled "50" is the frequency of values from 0 to 50. **b: RBC Glutathione Distribution 1-2 months after first round (3 days) of DMSA**. The distribution has tightened dramatically, so that most children are within the laboratory's reference range for typical adults. **c: Change in RBC glutathione 1-2 months after first round of DMSA**. Children with initially low levels had a large increase in glutathione, and children with high levels had a large decrease, so that most levels of glutathione normalized. The black dots are the participants who received DMSA and glutathione lotion and the red dots are the participants who received DMSA and the placebo lotion. The trendlines for the two groups lie directly on top of one another, suggesting that the effect of the glutathione lotion is negligible compared to the effect of the DMSA.

The level of glutathione in the Red Blood Cells (RBC) was measured at baseline and at the beginning of Phase 2 (for those who continued on to phase 2), which was typically 1.5 months (42 days +/- 33) after taking the DMSA. The values are given in Table 8 and Figure 4. Initially there was a very broad distribution of levels, with some being lower or higher than the lab's reference range for adults, defined as +/- 2 standard deviations around the mean. (It should be noted that children tend to have lower levels than adults, but the laboratory did not have a pediatric reference range.) After administration of DMSA, there was little change in the average level (a small decrease, not statistically significant), but the standard deviation became dramatically smaller. Figure 4c plots the average change in glutathione vs. the initial level. The change in glutathione level had an extremely high inverse correlation (-0.96, p < 0.0001) to the initial glutathione. Specifically, the children with unusually low initial levels had a large increase in glutathione towards the average value, those children with unusually high initial levels had a large decrease towards the average value, and those with initially average values had little change. Note that the change was very similar regardless of whether the participants were receiving the glutathione lotion or the placebo lotion, suggesting that the effect was due to the DMSA.

**Table 8:** Glutathione levels before and 1-2 months after 1^st ^round of DMSA in Phase 1 (just prior to Phase 2).

	**Average Glutathione Level**	**Range**

**Baseline - all (n = 72)**	501 +/- 246	31-1033
**Baseline - those with a 2**^**nd **^**measurement in phase 2 (n = 38)**	523 +/- 280	31-1033
**1-2 months after 1**^**st **^**round DMSA (n = 38)**	478 +/- 83	355-695
**Immunosciences Reference Range for Adults**		427-714

There is a dramatic change in the standard deviation.3

### RBC Glutathione and Heavy Metals

Correlation analysis found that baseline RBC glutathione (before DMSA) had significant positive correlations with urinary excretion of several metals (at baseline and after 9^th ^dose of DMSA), including baseline Sb (r = 0.26), and post-9th dose urinary excretion of Pb (r = 0.25), Al-9 (r = 0.29), and Cd (r = 0.30), suggesting that increased levels of these metals correlated with increased RBC glutathione. This suggests that the body responds to increased levels of most toxic metals by increasing the production of RBC glutathione. However, there was a negative correlation of RBC glutathione with excretion of mercury at the 9th dose (r = -0.27), which suggests that mercury has an adverse effect on RBC glutathione, presumably by both causing it to be excreted from the body after the glutathione binds to mercury, and perhaps more importantly by inhibiting production of glutathione. It is very interesting that mercury, and only mercury, has a significant negative correlation with RBC glutathione, and again suggests the special importance and toxicity of mercury.

A stepwise linear regression analysis for predicting initial glutathione was conducted based on the urinary excretion of toxic metals at baseline and after the 9^th ^dose of DMSA. The results are shown in Table 9. It was found that the initial glutathione could be partially predicted (adjusted R^2 ^= 0.25, p = 0.003) based primarily on excretion of cadmium, lead, tin, thallium, and arsenic.

**Table 9:** Regression analysis of initial glutathione and change in glutathione (from before and after phase 1).

	**Adjusted R**^**2**^	**P-Value**	**Equation**	**Most Significant variables**
**Glutathione (n = 59)**	0.25	0.003	378.7-48.8 PbB + 3.67AsB -219 CdB +2.49 Sn9 +369 TlB -69.9 Hg9 -4.32 As9 +598 Cd9	Cd9**, PbB*, Sn9*, Tl9*, As9*
**Change in glutathione (n = 37)**	0.29	0.002	104-2.26Sn9+87.8Hg9-669Cd9	Cd**

The analysis involved metals at Baseline (B) and after the 9^th ^dose (9).

A similar analysis was conducted for the change in glutathione between baseline and after the first round of DMSA, and it was found that the change in glutathione could be partially predicted (adjusted R^2 ^= 0.29, p = 0.002) based primarily on excretion of cadmium after the 9^th ^dose in phase 1 (see Table 9). This is consistent with the correlation analysis above.

### Complete Blood Count

Table 10 shows the results of the initial complete blood count for 77 participants, prior to any treatment. The only somewhat unusual feature at the initial blood draw was that 18% had elevated platelets (reference range of 130-450 k/mm^3^), a marker of inflammation. The % Lymphocytes tended to be slightly above or below the reference range, but the absolute lymphocytes were almost all within the reference range.

**Table 10:** Complete Blood Count and Chemistry Panel at baseline (N = 77).

	**Average Value**	**Stnd Dev**	**% Below RR**	**% Above RR**
**WBC (k/mm3)**	8.04	2.90	1%	9%
**RBC (m/mm3)**	4.56	0.29	4%	1%
**HEMOGLOBIN(g/dL)**	13.0	0.76	1%	3%
**Hematocrit (%)**	37.9	2.27	1%	1%
**MCV (fL)**	83.3	3.21	0%	4%
**MCH (pg)**	28.6	1.16	3%	3%
**MCHC (g/dL)**	34.4	0.73	0%	0%
**RDW (%)**	13.4	0.70	0%	4%
**Platelet count (k/mm3)**	388	208	1%	**18%**
**MPV (fL)**	8.53	1.02	8%	3%
**Seg Neutrophils**	44.5	12.6	8%	9%
**Lymphocytes (%)**	44.7	11.6	12%	13%
**Monocytes (%)**	7.15	1.91	0%	3%
**Eosinophils (%)**	3.42	2.55	0%	8%
**Basophils (%)**	0.41	0.52	0%	0%
**Abs Neutrophil (K/uL)**	3.80	2.35	0%	8%
**Abs lympocyte (k/uL)**	3.29	0.87	1%	0%
**Abs Monocyte (k/uL)**	0.55	0.21	0%	1%
**Abs Eosinophil (k/uL)**	0.24	0.17	0%	0%
**Abs Basophil (k/uL)**	0.02	0.04	0%	0%
**Glucose (mg/dL)**	87.9	14.9	3%	16%
**Urea Nitrogen (mg/dL)**	13.7	4.18	1%	0%
**Creatinine**	0.50	0.09	**22%**	0%
**Bun/Creat Ratio**	28.0	8.45	0%	**43%**
**Uric Acid (mg/dL)**	3.93	0.82	1%	4%
**Sodium (mmol/L)**	140	2.33	1%	1%
**Potassium (mmol/L)**	4.39	0.45	0%	4%
**Chloride (mmol/L)**	104	2.20	0%	0%
**CO2 (mmol/L)**	22.3	2.28	9%	0%
**Anion gap**	13.6	2.52	0%	3%
**Osmolality (mOs/kg)**	284	5.15	1%	1%
**Protein (g/dL)**	7.23	0.48	0%	3%
**Albumin (g/dL)**	4.57	0.21	0%	0%
**Globulin (g/dL)**	2.70	0.34	0%	1%
**Alb/Glob Rat**	1.73	0.23	0%	6%
**Cholesterol (mg/dL)**	150	25.2	0%	14%
**Triglyceride (mg/dL)**	100	62.2	0%	13%
**Calcium (mg/dL)**	10.0	0.37	0%	3%
**Phosphorus (mg/dL)**	5.04	0.61	0%	9%
**Alkaline Phosphate (IU/L)**	237	64.9	0%	8%
**GGT (IU/L)**	10.3	3.13	0%	1%
**ALT (IU/L)**	20.1	8.03	0%	4%
**AST (IU/L)**	35.2	8.45	0%	6%
**Lactic Dehydrogenase (IU/L)**	253	56.2	1%	6%
**Bilirubin (mg/dL)**	0.34	0.25	8%	0%

The major abnormalities are elevated platelet counts, low creatinine, and elevated BUN/creatinine ratio.

**Figure 5:**
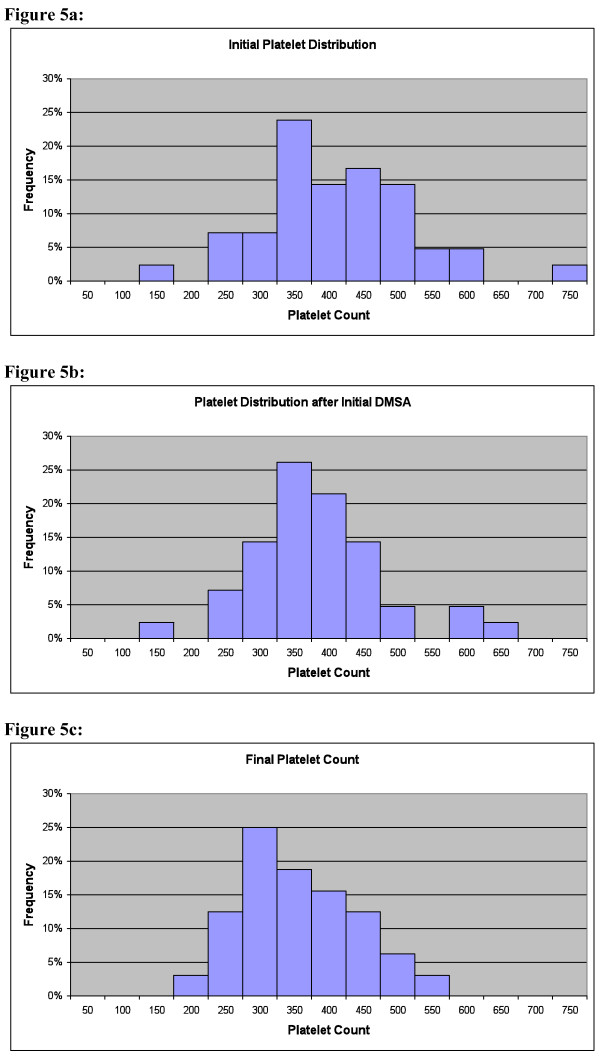
**Platelet count**. **a: Platelet Count before DMSA**. Note many children have values above the reference range (130-450 k/mm^3^). One child had a very high value (1996 k/mm^3^) which is not shown on this chart. **b: Platelet Count 1-2 months after first round of DMSA**. The primary effect of DMSA is to decrease the elevated platelets, with little effect on those in the normal or low range. One child still had a very high value (1917 k/mm^3^) which is not shown on this chart. **c: Platelet Count at End of Study**. Both the 7-round and 1-round groups are plotted together, as the results were similar, although there was slightly more decrease for the 7-round than the 1-round group. The primary effect of DMSA is to normalize elevated or (in one case) low platelets. One child still has a high value of 1195 k/mm^3 ^which is not shown, but it has decreased substantially from its initial value of 1996 k/mm^3^).

Table 11 shows the changes in Complete Blood Count (CBC), measured before and 1.5 months (42 +/- 33 days) after the first round of DMSA. The only significant change was a 6% decrease in platelet count (p = 0.02). The decrease in platelet count was primarily in the group with an initial level above the reference range (decrease of 70 k/mm^3^), whereas the group initially in the reference range had much less change (decrease of 24 k/mm^3^), and the one child with an initially low level had an increase of 26 k/mm^3 ^The correlation of the. initial platelet level and the change was -0.32, p = 0.05. Figure 5a shows the initial distribution of platelet count, Figure 5b shows the value 1-2 months after the first round of DMSA, and Figure 5c shows the values at the end of phase 2 for both groups. Overall, the many children with initially high levels had a decrease towards the normal range, and the one child with an initially low value had an increase towards the normal range. It is interesting to note that one child had an extremely high initial value of 1996 k/mm^3^, and it decreased by the end of the study to 1195 k/mm^3^.

**Table 11:** Complete Blood Count and Chemistry Panel at baseline and after 1 round of DMSA (N = 41).

	**Baseline Average Value**	**Stnd Dev**	**% Below RR**	**% Above RR**	**% change After 1**^**st **^**DMSA**	**% Below RR**	**% Above RR**
**WBC (k/mm3)**	7.76	2.50	0%	12%	-3%	5%	2%
**RBC (m/mm3)**	4.60	0.27	0%	2%	0%	0%	0%
**HEMOGLOBIN(g/dL)**	13.2	0.69	0%	2%	1%	0%	5%
**Hematocrit (%)**	38.5	2.05	0%	2%	0%	2%	0%
**MCV (fL)**	83.8	2.94	0%	2%	0%	0%	2%
**MCH (pg)**	28.7	0.98	0%	2%	0%	0%	2%
**MCHC (g/dL)**	34.3	0.72	0%	0%	0%	0%	0%
**RDW (%) Platelet count**	13.4	0.55	0%	0%	0%	0%	7%
**(k/mm3)**	424	274	**2%**	**29%**	**-7%**	**0%**	**12%**
**MPV (fL)**	8.59	1.14	10%	5%	1%	10%	2%
**Seg Neutrophils**	42.6	11.9	15%	2%	3%	20%	7%
**Lymphocytes (%)**	46.2	11.1	5%	17%	-4%	7%	22%
**Monocytes (%)**	7.27	2.01	0%	2%	5%	2%	7%
**Eosinophils (%)**	3.78	2.91	0%	10%	-1%	0%	10%
**Basophils (%) Abs Neutrophil**	0.39	0.54	0%	0%	25%	0%	0%
**(K/uL) Abs lympocyte**	3.34	1.53	0%	2%	2%	5%	5%
**(k/uL) Abs Monocyte**	3.28	0.86	0%	0%	0%	2%	0%
**(k/uL) Abs Eosinophil**	0.53	0.17	0%	0%	9%	0%	0%
**(k/uL)**	0.24	0.17	0%	0%	28%	0%	5%
**Abs Basophil (k/uL)**	0.01	0.03	0%	0%	140%	0%	0%
**Glucose (mg/dL) Urea Nitrogen**	85.3	12.5	5%	15%	-4%	5%	7%
**(mg/dL)**	13.9	4.18	2%	0%	-8%	0%	0%
**Creatinine**	0.50	0.08	**20%**	**0%**	**-5%**	**22%**	**0%**
**Bun/Creat Ratio**	28.4	9.40	**0%**	**39%**	**-8%**	**2%**	**32%**
**Uric Acid (mg/dL)**	3.73	0.80	2%	2%	13%	7%	0%
**Sodium (mmol/L)**	139	2.11	0%	0%	-2%	2%	2%
**Potassium (mmol/L)**	4.41	0.46	0%	5%	-2%	0%	10%
**Chloride (mmol/L)**	103	1.71	0%	0%	-2%	0%	0%
**CO2 (mmol/L)**	22.8	1.96	5%	0%	-5%	20%	0%
**Anion gap Osmolality**	13.3	2.42	0%	2%	2%	0%	5%
**(mOs/kg)**	284	4.83	0%	0%	12%	2%	2%
**Protein (g/dL)**	7.17	0.53	0%	0%	5%	0%	0%
**Albumin (g/dL)**	4.60	0.21	0%	0%	5%	0%	0%
**Globulin (g/dL)**	2.65	0.31	0%	2%	6%	0%	0%
**Alb/Glob Rat**	1.77	0.23	0%	12%	4%	0%	5%
**Cholesterol (mg/dL)**	147	30.1	0%	15%	19%	0%	29%
**Triglyceride (mg/dL)**	87.8	50.0	0%	7%	30%	0%	12%
**Calcium (mg/dL)**	10.1	0.37	0%	2%	-4%	0%	10%
**Phosphorus (mg/dL)**	5.09	0.67	0%	10%	4%	0%	12%
**Alkaline Phosphate (IU/L)**	228	64.1	0%	2%	7%	0%	20%
**GGT (IU/L)**	10.2	2.99	0%	0%	12%	0%	0%
**ALT (IU/L)**	20.2	7.29	0%	2%	7%	0%	5%
**AST (IU/L)**	34.4	5.54	0%	0%	3%	0%	0%
**Lactic Dehydrogenase (IU/L)**	243	43.2	2%	0%	14%	2%	15%
**Bilirubin (mg/dL)**	0.33	0.31	10%	0%	-12%	5%	2%

Data on platelets, creatinine, and BUN/creatinine is highlighted.

White blood cell count was investigated carefully, due to concerns about the possible effect of DMSA on it. For the 7-round group there was a 7% decrease in white blood cell count (not significant), with 1 child initially having a high level, and at the end 1 child having a high level and one child ending with a level slightly below the reference range (3.9 k/mm^3^, compared to a reference range of 4.0-12.0 k/mm^3^). The child who ended with a slightly low level had started with a level of 4.9 k/mm^3^; at the beginning of phase 2 it was 3.6, then in the middle of the study it was 5.2 k/mm^3^, so the total white blood cell count fluctuated near the bottom of the reference range during the study, but at no point was the participant either neutropenic or lymphocytopenic. For the 1-round group, there was an 8% decrease (not significant) in white blood cell count with one child initially having an elevated level, and all being normal at the end of the study.

A stepwise linear regression analysis of initial platelet count did not reveal any significant relationship with urinary excretion of toxic metals (at baseline and after 9^th ^dose) and initial glutathione. However, a similar analysis for the change in platelet count from beginning of phase 1 to the beginning of phase 2 was performed, with excretion of toxic metals after the 9^th ^dose of DMSA and change in glutathione as the dependent variables. It was found that the change in platelet count could be partially explained (adjusted R^2 ^= 0.41, p = 0.02), with the major factors being Tl (p = 0.002), As (p = 0.01), Cd (p = 0.03), and change in glutathione (p = 0.04). Table 12

**Table 12:** Stepwise linear regression analysis of change in platelet count (from baseline to beginning of phase 2, 1-2 months after DMSA therapy), based on excretion of metals after the 9^th ^dose in phase one, and the change in glutathione.

	**Adjusted R**^**2**^	**P- value**	**Equation**	**Most Significant Variables**
**Change in platelet count (both groups, n = 32)**	0.41	0.02	7rnd group (3.71-331Tl9 +256Sb9+2.89As9-256Cd9-0.118Dglut) 1rnd group (-81.6 +28.3Tl9- 174Sb9+0.194As9+259Cd9+0.193Dglut)	7rnd - (Tl9***, As9**, Cd9*, Dglut*), 1rnd- (Dglut*)
**Change in platelet count (7-rnd group only, n = 20)**	0.41	0.02	3.71 -331 Tl9 +256Sb9 +2.89 As9 -256 Cd9 -0.118 DGlut	Tl9**, As9**, Cd*, DGlut*

Thallium (Tl), Arsenic (As), Cadmium (Cd), and change in glutathione (Dglut) are the best predictors of change in platelet count, for the case of analyzing both groups and for the case of the 7-round group only.

### Chemistry Panel

Table 10 shows the results of the initial blood chemistry panel for 77 participants in Phase 1. The major unusual feature was the abnormally high Blood Urea Nitrogen (BUN)/Creatinine ratio, an indicator of kidney function. Initially, 43% of the children had a value above the laboratory's reference range, and none had a level below the reference range. This was largely due to low serum creatinine (22% of the children had levels below the reference range, and none had levels above it). The BUN values were almost all within the reference range, but tended to be in the higher end of it.

Table 11 also shows the changes in blood chemistry, measured before and 1.5 months (42 +/- 33 days) after the first round of DMSA. There were no statistically significant changes in blood chemistry. However, it is interesting to note that initially 17/42 participants had elevated Blood Urea Nitrogen (BUN)/Creatinine ratios, and after the DMSA it decreased 6% (not significant), so that only 14/42 had elevated levels, and 1/42 had low levels. The reason for the initial elevated ratio is largely due to low creatinine (in 8/42 children), whereas none had high initial BUN values. Low creatinine was defined as less than 0.4 (younger) or 0.5 (older) mg/dl. Overall, this suggests there was no worsening of kidney function.

Stepwise linear regression analysis found that the variations in initial BUN/Creatinine ratio could be partially predicted by urinary metal excretion at baseline and after the 9^th ^dose of Phase 1, with an adjusted R^2 ^= 0.35, p = 0.0001, with the most significant factors being baseline Sn (p = 0.003), baseline W (p = 0.02), and 9^th ^dose Sb (p = 0.05).

A similar analysis found that the changes in BUN/Creatinine from beginning of phase 1 to beginning of phase 2 could be partially predicted (adjusted R^2 ^= 0.51, p = 0.05, with the most significant factors being 9^th ^dose Hg (p = 0.01), change in glutathione (p = 0.02), 9^th ^dose Tl (p = 0.04), and 9^th ^dose Pb (p = 0.05).

At the end of phase 2, the 7-round treatment group did not have any significant changes in blood chemistry. There was a possible trend of a small decrease in eosinophils (-18%, p = 0.08), with initially 3/21 high, and only 2/21 high at the end of the study. There was also a possible trend of an increase in triglycerides (+47%, p = 0.06) - initially all were normal, and at the end of the study 2/21 were elevated. In contrast, in the 1-round treatment group, there was a 15% decrease (not significant) in triglycerides levels. It should be noted that these were not fasting measurements, so the fluctuations could be diet-related.

It is interesting to note that there was a 12% increase (not significant) in alkaline phosphatase, with initially 0/42 having abnormal levels, and 5/42 having elevated levels at the end of the study. Elevated alkaline phosphatase is associated with growth spurts, and it is expected that children in the study age range will normally have growth spurts.

### Heavy Metal Exposure Questionnaire

Table 13 lists the medical history data that relates to toxic metal exposure, as reported by the parents. Some of these factors were analyzed to determine if they correlated with severity of autism. Maternal fish consumption (a primary source of mercury) and total oral antibiotic use (which inhibits excretion of mercury) over the first 3 years of life did not significantly correlate with any of the autism severity scales at baseline. The number of maternal mercury amalgam fillings did not correlate with the SAS, PDD-BI autism scale or the ADOS total, but there was a negative correlation (-0.33) of the ATEC total with maternal mercury dental fillings - this is unexpected, as it suggests that a higher number of mercury fillings was associated with less severe autism.

**Table 13:** Medical History, as reported by parents (n = 73)

**Maternal Fish Consumption (servings/month)**	2.85 +/- 2.9
**Mercury Amalgams present during pregnancy**	3.12 +/- 3.5
**Mercury amalgams placed during pregnancy**	3%
**Mercury amalgams removed during pregnancy**	2%
**Placed during Nursing**	2%
**Removed during Nursing**	2%
**Antibiotic usage during pregnancy (rounds)**	0.67 +/- 1.5
**Antibiotic usage during nursing (rounds)**	0.39 +/- 0.83
**Child usage of oral antibiotics**	
**0-6 months**	0.76 +/- 1.2
**7-12 months**	1.63 +/- 2.9
**12-24 months**	2.04 +/- 3.0
**24-36 months**	1.82 +/- 2.0
**Total 0-36 months**	5.4 +/- 6.1
**Ear Infections 0-36 months**	3.5 +/- 4.3
**Blood Type**	33% A+, 4% A-, 11% B+, 2%B- 4%AB+, 22%O+, 22%O-
**Rhogam use**	18%
**Reactions to vaccines**	38% none, 44% mild, 10% moderate, 7% severe
**Eating/Licking Paint**	7% mild, 4% moderate, 5% severe
**Pica**	16% mild, 8% moderate, 16% severe
**Pregnancy Complications**	31% mild, 9% moderate, 9% severe
**Birth Complications**	23% mild, 11% moderate, 11% severe
**Childhood Illness**	10% mild, 4% moderate, 6% severe
**Regression**	28% no regression, 30% possible regression age 18.2 +/- 7.8 mo, 42% regression (age 20.8 +/- 16 mo

Table 14 shows the strong correlation of "eating/licking paint" and pica with the autism severity scores and with metal excretion. It should be noted that the correlation scores are generally quite similar for both the autism severity scales and for metal excretion.

**Table 14:** Correlations of symptoms of "Eating/licking paint" and "pica" with symptoms of autism and urinary excretion of metals (at baseline or immediately after the 9^th ^dose of DMSA in Phase 1).

	**Eating/Licking Paint**	**Pica**
**ATEC Total**	n.s.	0.33
**ADOS Total**	0.39	0.43
**SAS**	0.54	0.63
**PDD-BI - modified Autism subscale**	0.51	0.54
		
**Pb-b**	n.s.	0.32
**Pb-9**	0.62	0.40
**Sn-b**	0.35	0.34
**Tl-b**	0.29	0.38
**Tl-9**	0.39	0.33
**Sb-b**	0.49	0.45
**W-b**	0.31	0.45
**Al-9**	0.38	n.s.

Correlations of symptoms were checked for all toxic metals listed in table 2, but only the significant ones (r > 0.30) are listed below.

## Discussion

### Urinary Excretion - Toxic Metals

DMSA greatly increased the excretion of lead, which is not surprising since it is FDA-approved for treating lead poisoning. There were also large increases in tin and bismuth (which are less toxic), some increases in mercury, thallium, antimony, and tungsten, and possible increases in uranium and (in later rounds) nickel. There was generally little effect on cadmium, arsenic, or aluminum.

Only 20% of the children had their therapy stopped after round 3 of phase 2, due to a decrease in urinary excretion below our chosen criteria. In 80% of the cases the level of excretion of lead remained high even at the end of round 6 in phase 2, suggesting that more rounds were needed. When this study was designed, it was unknown how long to treat for, and this data suggests that longer treatment is needed by most children to further reduce levels of lead and other toxic metals. Other medications, such as DMPS, would be more effective in removing mercury. It appears that other medications would be needed to treat cadmium, arsenic, or aluminum toxicity, if present.

It is interesting to compare the results of this study with the study by Bradstreet et al. 2003 as in phase 1 we replicated their dosing (9 doses over 3 days, 10 mg/kg-dose). In that study, they did not measure the baseline urinary excretion, but they did measure urinary excretion after 9 rounds of DMSA, at the same dosage we used. They reported a higher excretion of lead after 9 rounds of DMSA (18.2 +/- 43.3 vs. 9.2 +/- 7.8 mcg/g-creatinine). They found much higher excretion of mercury (6.4 +/- 13 vs. 0.97 +/- 0.88 mcg/g-creatinine) and slightly higher excretion of cadmium (0.48 +/- 0.42 vs. 0.33 +/- 0.23). Samples were collected in a similar fashion in both studies, and the measurements were done by the same laboratory, so a collection or measurement error is unlikely. Rather, it appears that the population in the Bradstreet et al study had a higher body burden of lead, mercury, and cadmium, perhaps because they were slightly older (6.2 yr, range 3 to 15 years) or more likely because they were from a different area of the country (across the US vs. Arizona). Unlike most of the rest of the US, Arizona has had unusually rapid population growth over the last several decades, from 2.7 million in the 1970 census to an estimated 6.1 million in 2006. Thus, Arizona had much less use of lead paint and leaded gasoline than other parts of the country, and that may account for the difference in lead levels between this study and the Bradstreet study. The difference in mercury levels between this study and Bradstreet's study may have similar origins due to geographical differences in diet (seafood) and environmental exposure. Therefore, it is important that geographical effects be considered when comparing toxic metal exposure from different studies.

### Urinary Excretion of Essential Minerals

Based on our measurements, DMSA does have a significant effect on urinary excretion of several essential minerals. However, in most cases the amount of loss is only a small fraction (less than 6%) of the RDA, and hence is of minor concern. The two exceptions were potassium and chromium, which had an average extra daily loss of 57% and 88% of the RDA, during the 3 days of DMSA. However, since there were at least 11 days between each round of DMSA, the effective loss over 2 weeks is much less. The blood measurements of potassium revealed no change over the course of the study, so the children's bodies appeared to be able to compensate for minor loss of potassium. Since the children were on a multi-vitamin/mineral supplement of their choice (most of which included chromium), the loss of chromium was probably not a problem. In general, our data suggests that people using DMSA should take a chromium supplement, and consume extra fruits and vegetables (for potassium, which is not allowed in significant levels in over-the-counter multi-vitamin/mineral supplements).

Since the DMSA was taken orally, and since only about 20% is absorbed orally, it is possible that some of the 80% remaining in the digestive tract bound to essential minerals in food, causing a decreased absorption of essential minerals. A future study could check red blood cell levels of essential minerals, to check for long-term effects, especially on chromium.

### Glutathione

DMSA treatment had a dramatic effect on normalizing abnormal levels of RBC glutathione. At the start of the study, some children had unusually high levels, and the correlation analysis suggests this was partially due to increased production in response to higher levels of toxic metals. Some children had unusually low levels, and correlation analysis suggests this was partially due to the presence of mercury, which is known to strongly inhibit the production of glutathione. Surprisingly, a single round of DMSA resulted in a dramatic normalization of the abnormal levels, and the effect lasted at least 1.5 months. The effect was the same for the group receiving the glutathione or placebo lotion, so the effect appeared to be due to the DMSA, not the glutathione lotion. It is possible that the DMSA's effect was partially due to its antioxidant capability, but it seems unlikely that that effect would be long-lasting. Instead, it seems likely that the DMSA's effect was due to the reduction of toxic metals in the RBC, allowing glutathione to return to a normal level. Since only 1 round was needed to normalize the RBC glutathione, and since the effect lasted at least 1.5 months, additional DMSA probably would have little additional benefit. Since glutathione serves many important functions in the body, improvements in glutathione might result in improvements in overall functioning, which suggests that children receiving 1 or 7 rounds of DMSA would have similar improvements in behavior. Table 15

**Table 15:** Correlation of initial glutathione with urinary excretion of toxic metals.

	**Baseline**	**1st dose**	**9th dose**
**Cd**	-0.08	0.21	0.30
**Al**	0.17	0.21	0.29
**Pb**	0.20	0.18	0.25
**W**	0.14	-0.05	0.23
**Sb**	0.26	0.22	0.22
**Sn**	-0.04	0.11	0.21
**U**	0.11	0.25	0.20
**Tl**	0.15	0.06	0.18
**Bi**	-0.07	0.09	0.12
**Ni**	-0.08	0.00	0.10
**As**	0.06	-0.04	-0.10
**Hg**	0.17	-0.30	-0.27

Arranged in order of degree of correlation with 9^th ^dose.

### Complete Blood Count

It was surprising that, at the start of the study, many of the children had elevated platelet levels. Platelet counts are an acute-phase reactant and an elevation is considered an indication of inflammation somewhere in the body. The platelet count did not significantly correlate with gut problems, so the inflammation does not appear to be primarily gut-related. It was more surprising that 1 round of DMSA resulted in a normalization of the platelet counts in about half of the cases, and the effects seemed to last for at least several months.

This suggests that some of the inflammation in children with autism is due to toxic metals which are removable by DMSA.

There was one case of 1 child whose White Blood Cell (WBC) count started near the bottom of the reference range, and it fluctuated above and below that value during the study, finally ending with a level slightly below the reference range, but without neutropenia or lymphocytopenia. So, although more investigation of effect of DMSA on WBC is needed, here does not appear to be a major detrimental effect of t DMSA on WBC.

### Chemistry Panel

There have been some reports that DMSA can slightly increase liver enzymes in 6-10% of people when used to treat children with lead poisoning (PDR 2008), which typically involves 21 continuous days of DMSA therapy at daily doses similar to that used in our study. However, we found that the DMSA had no significant effects on liver transaminases (AST and ALT), perhaps because we treated for only 3 days at a time, with 11 days in-between.

The only possible concern about the effect of the DMSA was a possible increase in triglyceride levels in the 7-round group. Triglyceride levels are best measured with a fasting blood draw, but we did not require fasting to simplify the ease of participation in the protocol. So, it is unclear if this result is a random artifact associated with non-fasting blood draws, or a real effect - future studies should check for this with a fasting blood draw.

We also found that many children had initially elevated BUN/creatinine ratio, which was most often due to low creatinine. The low creatinine is probably an indication of low muscle mass in the children. The variations in elevated BUN/creatinine could be partially explained with a regression analysis based on urinary excretion of heavy metals. This suggests that the elevations were partially due to the presence of heavy metals, especially tin, tungsten, and antimony. There was a small improvement in BUN/creatinine ratio by the end of the study, but the improvement was not statistically significant. There was no significant worsening of renal function of any study participant.

Overall, it appeared that the DMSA was well-tolerated.

### Adverse Effects

Most participants in the study reported overall improvements in their children. However, during Phase 1 there was one report of a mild adverse reaction (lethargy, decreased appetite), and that family chose not to continue to Phase 2. Also, duringPhase 2, 4 children dropped out due to adverse effects, with 2 of those children being on DMSA, and 2 being on placebo. There were also two children on DMSA who ended the study early because of low urinary excretion of metals, and they had some worsening of some symptoms; one had moderate sleep problems, and one had moderate sleep problems and increased tantrums. These problems were generally temporary, and resolved when treatment was stopped. On average, according to the Parental Global Impressions, there was little change (slight improvement) in tantrum behavior, and slight worsening of sleep and hyperactivity, so those are symptoms that should be documented in future treatment studies and may indicate possible temporary side-effects due to DMSA.

Note that the treatment protocol of 3 days of treatment followed by 11 days off is much lower than that recommended in the Physician Desk Reference for moderate/severe lead poisoning (21 days of continuous treatment), so it is expected that the treatment regimen used in this study would have fewer side-effects than for a 21-day treatment regimen. Overall, DMSA therapy in this study seemed relatively safe, with only a few reports of adverse effects that were generally temporary and of mild or moderate severity.

### Medical History

There was a very high correlation between "eating/licking paint" and pica and the severity of autism (up to r = 0.63 for SAS). The strong correlation of "eating/licking paint" and pica with urinary excretion of some metals (especially lead, tin, thallium, antimony, and tungsten) suggests that pica may be a significant source of toxic metals, accounting for up to half of the children's exposure to them. This may be part of a vicious cycle, where a worsening of autism leads to unusual behaviors including pica, which in turn leads to increased consumption of toxic metals and a worsening of symptoms.

### Limitations of the Study

1) The initial 3-day round of DMSA in Phase 1 was intended as a screening for Phase 2, but it was discovered that the initial round of DMSA had a large beneficial effect in dramatically improving glutathione, and normalizing platelet levels (a marker of inflammation). Thus, this study is best interpreted as a comparison of 1 round vs. 7 rounds of DMSA. A future study of 1 round vs. 0 rounds would be interesting.

2) At the end of Phase 2, 80% of the children were still excreting high amounts of lead and other toxic metals. Thus, it appears that most of the children would need a longer trial of DMSA to reduce their toxic metal levels.

3) Most of the metals had one or more correlations with other metals, making it difficult to distinguish the effect of one metal from another when doing regression analysis.

4) No measurement of plasma cysteine levels was conducted; since each molecule of DMSA is usually excreted in the urine bound to 2 molecules of cysteine, it is possible that DMSA therapy may decrease cysteine levels. However, since cysteine is a precursor of glutathione, and since glutathione levels normalized after 1 round of DMSA, this may not be a problem.

5) This study was large enough for phase 1, and large enough for phase 2 since the 1 round and 7 round groups could be combined for a regression analysis of changes in autism severity scores. However, a larger study is needed in future for a true placebo-controlled, double-blind study.

6) There was no measurement of urinary porphyrins, a marker for heavy metal toxicity. This would be very interesting to measure in a future study.

7) For statistical analyses involving multiple comparisons, care should be taken in determining which p-values are most significant. In general, for multiple comparisons we have assumed that p < 0.01 suggests that the results are significant.

## Conclusion

1) DMSA therapy, at the dosage and frequency in this study, is a relatively safe way to remove toxic metals, normalize glutathione, and improve elevated platelet levels and therefore possibly reduce inflammation in children with autism.

2) DMSA does cause significant increased urinary loss of potassium and chromium on the days it was taken, so modest supplementation of chromium and potassium (or increased intake of fruits and vegetables) may be helpful.

3) One round (3 days) of DMSA was nearly as effective as seven rounds of DMSA (3 days of DMSA, 11 days no dosing, repeated 7 times) in improving platelet levels.

4) In 20% of the participants, 3 rounds of DMSA was sufficient to lower excretion of toxic metals (primarily lead), but 80% of children were still excreting high levels of toxic metals (primarily lead) after 7 rounds. This suggests that longer treatment might be useful in reducing toxic metals in 80% of the children.

Future studies should consider longer treatment periods, and the use of other chelators. Pre-screening for metal toxicity using urinary porphyrins might be useful.

## Competing interests

The authors declare that they have no competing interests.

## Authors' contributions

JBA was the primary organizer of the study, did the initial data analysis, and was the primary author. He was the official co-principal investigator for the study. MB was the primary physician for the greater Phoenix area. He was the official principal investigator, and worked with JBA on study design and study approval. EG was the study coordinator and lead study nurse. JM was a secondary physician who assisted MB. JI was the ADOS evaluator for the greater Phoenix area. AH was a study nurse. IZ was the ADOS evaluator for the greater Tucson area. SN was the physician for the greater Tucson area. EG handled data entry and helped with data analysis. RAR was the primary statistician for the study, and did the regression analysis. KM was the pharmacist who assisted with compounding the DMSA. JB was a physician who served as consultant for the study. JME was a physician who served as a consultant for the study. All authors read and approved of the final manuscript.

## Pre-publication history

The pre-publication history for this paper can be accessed here:



## References

[B1] Ballatori N, Clarkson TW (1985). Biliary secretion of glutathione and of glutathione-metal complexes. Fundam Appl Toxicol.

[B2] Ballatori N, Clarkson TW (1984). Dependence of biliary secretion of inorganic mercury on the biliary transport of glutathione. Biochem Pharmacol.

[B3] Rowland IR, Robinson RD, Doherty RA (1984). Effect of diet on mercury metabolism and excretion in mice given methylmercury: role of gut flora. Arch Env Health.

[B4] James SJ, Cutler P, Melnyk S, Jernigan S, Janak L, Gaylor DW, Neubrander JA (2004). Metabolic biomarkers of increased oxidative stress and impaired methylation capacity in children with autism. Am J Clin Nutr.

[B5] James SJ, Melnyk S, Jernigan S, Cleves MA, Halsted CH, Wong DH, Cutler P, Bock K, Boris M, Bradstreet JJ, Baker SM, Gaylor DW (2006). Metabolic endophenotype and related genotypes are associated with oxidative stress in children with autism. Am J Med Genet B Neuropsychiatr Genet.

[B6] Audhya T Nutritional Abnormalities in children with Autism. AutismOne Conference in on May 2004 in Chicago, IL.

[B7] Konstantareas MM, Homatidis S (1987). Ear infections in autistic and normal children. J Autism Dev Disord.

[B8] Adams JB, Holloway CE, Margolis M, George F Heavy Metal Exposures, Developmental Milestones, and Physical Symptoms in Children with Autism. Conference Proceedings of the Fall 2003 Defeat Autism Now! Conference on Oct 3-5, 2003 in Portland, Oregon.

[B9] Adams JB, Romdalvik J, Ramanujam VMS, Legator MS (2007). Mercury, lead, and zinc in baby teeth of children with autism vs. controls. J Toxicology Environ Health.

[B10] Adams JB, Romdalvik J, Levine KE, Hu L-W (2008). Mercury in First-Cut Baby Hair of Children with Autism vs. Typically-Developing Children. Toxicological and Environmental Chemistry.

[B11] Rowland IR, Davies M, Evans J (1980). Tissue content of mercury in rats given methylmercury chloride orally: Influence of intestinal flora. Arch Environ Health.

[B12] Rowland IR, Grasso P, Davies MJ (1975). The methylation of mercuric chloride by human intestinal bacteria. Experientia.

[B13] Holmes AS, Blaxill MF, Haley BE (2003). Reduced Levels of Mercury in First Baby Haircuts of Autistic Children. Int J Toxicology.

[B14] Kern JK, Grannemann BD, Trivedi MH, Adams JB (2007). Sulfhydryl-reactive metals in autism. J Toxicol Environ Health A.

[B15] Ip P, Wong V, Ho M, Lee J, Wong W (2004). Mercury exposure in children with autistic spectrum disorder: case-control study. J Child Neurol.

[B16] DeSoto MC, Hitlan RT (2007). Blood Levels of Mercury are Related to Diagnosis of Autism: A Reanalysis of an Important Data Set. J Child Neurology.

[B17] Adams JB, Holloway CE, George F, Quig D (2006). Toxic metals and essential minerals in the hair of children with autism and their mothers. Biol Trace El Res.

[B18] Bradstreet J, Geier DA, Kartzinel JJ, Adams JB, Geier MR (2003). A Case-Control Study of Mercury Burden in Children with Autistic Spectrum Disorders. J Am Phys Surg.

[B19] Geier DA, Geier MR (2003). An assessment of the impact of thimerosal on childhood neurodevelopmental disorders. Pediatr Rehabil.

[B20] Geier DA, Geier MR (2004). A comparative evaluation of the effects of MMR immunization and mercury doses from thimerosal-containing childhood vaccines on the population prevalence of autism. Med Sci Monit.

[B21] Geier DA, Geier MR (2004). Neurodevelopmental disorders after thimerosal-containing vaccines: a brief communication. Exp Biol Med (Maywood).

[B22] Geier DA, Geier MR (2005). A two-phased population epidemiological study of the safety of thimerosal-containing vaccines: a follow-up analysis. Med Sci Monit.

[B23] Geier DA, Geier MR (2006). A meta-analysis epidemiological assessment of neurodevelopmental disorders following vaccines administered from 1994 through 2000 in the United States. Neuro Endocrinol Lett.

[B24] Geier DA, Geier MR (2006). A prospective assessment of porphyrins in autistic disorders: a potential marker for heavy metal exposure. Neurotox Res.

[B25] Geier DA, Geier MR (2006). An assessment of downward trends in neurodevelopmental disorders in the United States following removal of thimerosal from childhood vaccines. Med Sci Monit.

[B26] Geier DA, Geier MR (2006). An evaluation of the effects of thimerosal on neurodevelopmental disorders reported following DTP and Hib vaccines in comparison to DTPH vaccine in the United States. J Toxicol Environ Health.

[B27] Geier DA, Geier MR (2006). Early downward trends in neurodevelopmental disorders following removal of thimerosal-containing vaccines. J Am Phys Surg.

[B28] Geier MR, Geier DA (2003). Neurodevelopmental disorders after thimerosal-containing vaccines: A brief communication. Exp Biol Med.

[B29] Geier MR, Geier DA (2003). Thimerosal in childhood vaccines, neurodevelopment disorders, and heart disease in the United States. J Am Phys Surg.

[B30] Andrews N, Miller E, Grant A, Stowe J, Osborne V, Taylor B (2004). Thimerosal exposure in infants and developmental disorders: a retrospective cohort study in the United kingdom does not support a causal association. Pediatrics.

[B31] Fombonne E, Zakarian R, Bennett A, Meng L, McLean-Heywood D (2006). Pervasive developmental disorders in Montreal, Quebec, Canada: prevalence and links with immunizations. Pediatrics.

[B32] Hviid A, Stellfeld M, Wohlfahrt J, Melbye M (2003). Association between thimerosal-containing vaccine and autism. JAMA.

[B33] Madsen KM, Lauritsen MB, Pedersen CB, Thorsen P, Plesner AM, Andersen PH, Mortensen PB (2003). Thimerosal and the occurrence of autism: negative ecological evidence from Danish population-based data. Pediatrics.

[B34] Stehr-Green P, Tull P, Stellfeld M, Mortenson PB, Simpson D (2003). Autism and thimerosal-containing vaccines: lack of consistent evidence for an association. Am J Prev Med.

[B35] Verstraeten T, Davis RL, DeStefano F, Lieu TA, Rhodes PH, Black SB, Shinefield H, Chen RT (2003). Safety of thimerosal-containing vaccines: a two-phased study of computerized health maintenance organization databases. Pediatrics.

[B36] Bernard S, Enayati A, Roger H, Binstock T (2001). Autism: a novel form of mercury poisoning. Med Hypotheses.

[B37] Nelson KB, Bauman ML (2003). Thimerosal and Autism?. Pediatrics.

[B38] Agency for Toxic Substances and Disease Registry (1999). Toxicological Profile for mercury. section 16.

[B39] Nataf R, Skorupka C, Amet L, Lam A, Springbett A, Lathe R (2006). Porphyrinuria in childhood autistic disorder: implications for environmental toxicity. Toxicol Appl Pharmacol.

[B40] Geier DA, Geier MR (2006). A Prospective Assessment of Porphyrins in Autistic Disorders: A Potential Marker for Heavy Metal Exposure. Neurotoxicity Research.

[B41] Geier DA, Geier MR (2007). A Prospective Study of Mercury Toxicity Biomarkers in Autistic Spectrum Disorders. J Toxicology and Environmental Health.

[B42] Geier DA, Kern JK, Garver CR, Adams JB, Audhya T, Nataf R, Geier MR (2009). Biomarkers of environmental toxicity and susceptibility in autism. J Neurol Sci.

[B43] Stangle DE, Smith DR, Beaudin SA, Strawderman MS, Levitsky DA, Strupp BJ (2007). Succimer chelation improves learning, attention, and arousal regulation in lead-exposed rats but produces lasting cognitive impairment in the absence of lead exposure. Environ Health Perspect.

[B44] Stein J, Schettler T, Wallinga D, Valenti M (2002). In harm's way: toxic threats to child development. J Dev Behav Pediatr.

[B45] Adams JB, Baral M, Geis E, Mitchell J, Ingram J, Hensley A, Zappia I, Newmark S, Gehn E, Rubin RA, Mitchell K, Bradstreet J, El-Dahr JM (2009). Safety and Efficacy of Oral DMSA Therapy for Children with Autism Spectrum Disorders: Part B - Behavior Results. BMC Clinical Pharmacology.

[B46] Adams JB, Baral M, Geis E, Mitchell J, Ingram J, Hensley A, Zappia I, Newmark S, Gehn E, Rubin RA, Mitchell K, Bradstreet J, El-Dahr JM (2009). The Severity of Autism Is Partially Explained by Toxic Metal Body Burden and Red Blood Cell Glutathione Levels. Journal of Toxicology.

